# Individuals with long-term illness, disability or infirmity are more likely to smoke than healthy controls: An instrumental variable analysis

**DOI:** 10.3389/fpubh.2022.1015607

**Published:** 2023-01-09

**Authors:** Xingzuo Zhou, Yiang Li, Tianning Zhu, Yiran Xu

**Affiliations:** ^1^Institute for Global Health, University College London, London, United Kingdom; ^2^Department of Sociology, University of Chicago, Chicago, IL, United States; ^3^Department of Social Policy, London School of Economics and Political Science, London, United Kingdom; ^4^Centre of Development Studies, University of Cambridge, Cambridge, United Kingdom

**Keywords:** smoking, long-term illness or disability, causal inference, instrumental variable (IV), public health, simultaneous effect

## Abstract

Despite the prevalence of smoking cessation programs and public health campaigns, individuals with long-term illness, disability, or infirmity have been found to smoke more often than those without such conditions, leading to worsening health. However, the available literature has mainly focused on the association between long-term illness and smoking, which might suffer from the possible bidirectional influence, while few studies have examined the potential causal effect of long-term illness on smoking. This gap in knowledge can be addressed using an instrumental variable analysis that uses a third variable as an instrument between the endogenous independent and dependent variables and allows the identification of the direction of causality under the discussed assumptions. Our study analyzes the UK General Household Survey in 2006, covering a nationally representative 13,585 households. We exploited the number of vehicles as the instrumental variable for long-term illness, disability, or infirmity as vehicle numbers may be related to illness based on the notion that these individuals are less likely to drive, but that vehicle number may have no relationship to the likelihood of smoking. Our results suggested that chronic illness status causes a significantly 28% higher probability of smoking. The findings have wide implications for public health policymakers to design a more accessible campaign around smoking and for psychologists and doctors to take targeted care for the welfare of individuals with long-term illnesses.

## 1. Introduction

It is well-documented that smokers have poorer physical and mental health than non-smokers, as measured by several subjective and objective indicators (1, 2). Numerous studies have examined the link between cigarette smoking and certain types of illnesses, impairments, morbidity, and low health-related quality of life, suggesting that smokers are more likely to get lung cancers, cardiovascular diseases, and low subjective health than non-smokers (3, 4). The present research also indicates that individuals with long-term illness, disability, or infirmity (LSI), defined as conditions for chronic sickness in the 2006 British General Household Survey, could be more prone to be smokers and less likely to undergo smoking cessations or screenings for tobacco use than healthy people (5–7).

From a psychological perspective, high levels of substance abuse could be attributed to the higher subjective utility smokers receive relative to the perceived health drawbacks of tobacco consumption (8–10). Specifically, for people with LSI, smoking is a common strategy to cope with the social isolation they face alongside the mental health issues that come with it (11, 12). In the US, people with LSI had a substantially higher smoking rate than the national average of 12.5% in 2020. Nearly 20 of every 100 American adults with a physical disability (19.8%) are smokers, whereas only 11.8% of their counterparts without disabilities smoke (13). Beyond the descriptive statistics, recent research has empirically examined the smoking prevalence in persons with activity limitations using a regression framework (14, 15). For example, using logistic regression on cross-sectional survey statistics in the US, Fitzmaurice et al. (14) found that individuals who reported mobility limitations were more likely to be overweight and to become smokers. The other study confirmed the finding that disabled Americans smoked more cigarettes per day than people without disabilities and also found that they were less able to fully participate in all preventive services offered (16). Similar results were found in the UK. British adults with physical impairments smoke 20 or more cigarettes per day than their counterparts while holding age and gender constant (17). Other than the Western evidence on smoking addiction among disabled people, previous studies suggest a similar effect in Asia. Lee et al. (18) revealed that people South Koreans with physical impairments listed on the National Disability Registration System had higher smoking rates and were less likely to quit smoking than the general population in South Korea.

In brief, the literature reviewed above suggests a correlation that individuals with physical disabilities have higher smoking rates than healthy individuals. Such association has been found in various geographical locations and across cultural contexts. Nonetheless, existing studies fail to address reverse causality. Reverse causality is when there is a bidirectional relationship between the dependent and independent variables. Smoking has been linked to the deterioration of people's physical condition and consequently restricts individuals' daily activity (16, 19, 20). Conversely, individuals' mobility-restricting conditions might also trigger smoking behavior, which leads to a bidirectional association (21). When ignoring reverse causality, the estimated correlation might be biased (22, 23). Moreover, with limited covariates included in the studies reviewed above, there might be Omitted Variable Bias (OVB) when the model fails to control for any variable that is correlated with both outcome and independent variable. With the presence of the OVB, the regression estimate will be systematically biased (24). Hence, the correlations suggested by the studies reviewed above revealed does not imply a causal link between physical disability and smoking.

Our research aims to examine the causal relationship between LSI and smoking, namely whether LSI people are more likely to smoke. Identifying causal links between long-term sickness or disability and smoking addiction is an important issue. If smoking is the cause, the smoking itself could be the primary target of effective intervention; conversely, if smoking is the result, it may be more effective for smoking cessation programs to target the identified causes, including LSI. Individuals with chronic diseases or disabilities face numerous obstacles to obtaining smoking cessation programs and other public health campaigns (25). These obstacles include lack of patient adherence, accessibility (e.g., transportation), and knowledge on how to accommodate people with disabilities, infirmity, and long-term illness, some of which may not be visible (26, 27). Hence, although individuals with disabilities have a smoking prevalence that is 50% higher than that of the general population, the types of tobacco cessation measures offered were not available to more than 40% of smokers with impairments who had sought assistance (6).

Using the instrumental variable approach, we aim to fill the current literature gap and study if individuals with LSI are more likely the current smokers. The instrumental variable method has been widely used in economics to evaluate causal effects when reverse causality is present (28, 29). A variable is exogenous if it does not correlate with unobserved factors in the regression and is endogenous if such a correlation exists. The instrument is an exogenous variable that correlates with the potentially observed endogenous regressor and only influences the dependent variable through the potentially endogenous regressor. In other words, the instrumental variable only impacts the response variable indirectly through an endogenous variable, and the response variable has no relationship with the instrumental variable (30, 31). As such, it avoids the bidirectional problem in the current literature studying the associations between chronic illness and smoking (14–16). Herein, we implemented the instrumental variable approach with the number of vehicles owned as an instrumental variable. The vehicle numbers can be related to LSI based on the notion that these individuals are less likely to drive; meanwhile, those vehicle numbers have no relationship to the likelihood of smoking after controlling a range of social demographic factors (32). We hypothesize that individuals with long-term illness, disability, or infirmity are more likely to smoke compared to those healthy ones.

## 2. Materials and methods

### 2.1. Data sources

The data used in this study were collected from the General Household Survey (GHS), which is a repeated cross-sectional study that was conducted annually (except for 1997–1998 and 1999–2000 due to significant survey redevelopment) by the Office for National Statistics in the UK starting in 1971. The GHS is now known as the General Lifestyle Survey after it was renamed in 2008 and incorporated into the Integrated Household Survey. The GHS aims to provide different government departments and organizations with data regarding an array of characteristics of British private households for monitoring and policy purposes, as well as present a general picture of the lives of people in the UK. The factors collected by the survey include both general private household information and individual traits. General private household information such as the number of household members, while individual traits consist of factors such as education, individual income, and marital status. For the current analysis, we used cross-sectional data collected for the GHS in 2006, the latest GHS available for access. A sample of ~13,585 households was randomly selected, and interviews were subsequently conducted with all adults aged 16 and above in every responding household (33). A total of 9,731 households consisting of 22,924 individuals responded to the interview invitation (including full and partial interviews) for a response rate of 73.1%. 5,666 samples are excluded from analysis because they are less than 18 years old. In the United Kingdom, a person must be 18 or older to purchase cigarettes, hence smoking-related questions are not given to anyone under the age of 18. Then we adopted the complete case analysis on remaining 17,258 observations by removing 3,925 ones with missing values for any selected variables. We have a final sample size of 12,297 from the GHS in 2006, which is equivalent to 78% of the original sample.

A total of 12 relevant variables of household and individual characteristics were chosen from the GHS in 2006 for the purpose of this study, with a focus on three particularly important variables. First, the outcome is a binary variable that indicates whether an individual currently smokes, denoted by **smoking**. This measure was coded as one if an individual was a smoker and a value of zero otherwise. Second, our independent variable of interest, **illness**, was coded as one if an individual was suffering from chronic sickness (long-term illness, disability, or infirmity) that limits an individual's daily activity and a value of zero otherwise. Third, the instrumental variable is the number of vehicles (cars and vans only) owned by the household where the individual is from is denoted by the **number of vehicles**. Nine other variables are demographic and socioeconomic factors, including **age**, **sex**, **marital status**, **number of family units in the household**, **minority**, **natural log of individual income**, **natural log of household income**, **socioeconomic group, and education level**. These variables are used as control variables in the econometric analysis in a later section. Among these variables, it is important to note that age, socioeconomic group, marital status, and education all consist of more than one binary indicator to identify different groups within a single variable; that is, they are factor variables. We also present the analysis stratified by the factor variables.

The summary of descriptive statistics is presented in Table 1. Among the 12,297 individuals, ~40.7% suffered from long-term illness, disability, or infirmity. Given that the variable of sex takes the value of one if an individual is male and takes the value of two if an individual is female, it can be seen from the descriptive data that ~54.5% of the sample in this study were female. In addition, 4.8% of the sample were considered ethnic minorities (i.e., non-white) in the UK. In terms of the proportion of individuals who smoked, the data from the GHS in 2006 indicated that 20.6% of the sample were current smokers, though the average smoking rate decreased to around 14% in 2020 (34). Finally, the individuals from the sample owned an average of at least 1.4 vehicles, and the mean of family units in the household was 1.08.

**Table 1:** Summary of descriptive statistics.

		**Observations**
**Smoking**		
Smoker	20.6%	2,528
Non-smokers	79.4%	9,769
**Illness**		
People with illness	40.7%	5,002
People without illness	59.3%	7,295
**Sex**		
Male	45.4%	5,587
Female	54.6%	6,710
**Minority**		
Non-white	4.8%	590
White	95.2%	11,707
**Education**		
No qualification	27.2%	3,346
O-level or equivalent	27.9%	3,428
A-level or equivalent	13.7%	1,688
Degree	31.2%	3,835
**Number of vehicles (at least owned)**		
Mean	1.40	
Standard deviation	0.87	
**Number of family units in household**		
Mean	1.08	12,297
Standard deviation	0.32	12,297
**ln (individual income)**		
Mean	10.04	12,297
Standard deviation	1.13	12,297
**ln (household income)**		
Mean	10.75	12,297
Standard deviation	1.03	12,297
**Marital status**		
Married/cohabiting/same-sex	69.2%	8,514
Single	13.7%	1,679
Widowed	8.2%	1,008
Divorced/separated	8.9%	1,096
**Socioeconomic group**		
Employers: Large	0.1%	17
Managers: Large	6.5%	801
Employers: Small	1.6%	197
Managers: Small	5.1%	621
Profit: Self-employment	1.0%	123
Profit: Employee	4.7%	576
Non-manual anchorman	18.5%	2,275
Non-manual foreman	3.9%	485
Junior non-manual	19.7%	2,420
Personal service	3.6%	441
Manual: Foreman	5.6%	693
Skilled manual	7.1%	879
Semiskilled manual	11.0%	1,347
Unskilled manual	5.0%	617
Own account non-profit	5.6%	691
Farmers: Employers & managers	0.1%	12
Farmers: Own account	0.4%	46
Agricultural workers	0.5%	56

Before quantitatively analyzing the causal impact of LSI on the possibility of smoking, we note in Table 2 that the proportion of smoking does not differ significantly according to the mean and odds ratio. However, one could not draw a causal conclusion from the raw difference in means or codes ratios due to the presence of the reverse causality and OVB we discussed earlier in the introduction section. Hence, an instrumental variable approach will be applied in the later section to determine the causality between **illness** and **smoking**.

**Table 2:** Proportion of smoking individuals with respect to illness status.

	**Illness**	
	**Yes**	**No**
Number of individuals	5,002	7,295
Proportion of smoking individuals	20.15%	20.84%
Standard deviation	40.11%	40.61%
Odds ratio (95% CI)	0.96 (0.88–1.05)	

### 2.2. Methods

We aimed to investigate the causal effect of whether individuals with LSI were more likely to smoke than those without such conditions. By controlling for a range of demographic, socioeconomic, educational, medical and family-related control variables, the following model is first proposed. For each individual i,


(1) $$\begin{array}{l} {Smoking}_{i}=c_{1}+\alpha_{1}Illness_{i}+\beta_{1}X_{i}^{{}^{\prime }}+\epsilon_{1,i} \end{array}$$

where **smoking** is the binary variable of smoking status in 2006; **illness** indicates whether an individual suffers from long-term illness, disability or infirmity; *c*_1_ is a constant; and X' denotes the set of controls. α_1_ is expected to identify the effect of long-term illness, disability, or infirmity on smoking. This generic ordinary least squares (OLS) framework has previously been the most common choice in similar studies. Our choices of control variables include both demographic and socioeconomic controls based on similar studies (35), and are shown below:

- age: a factor variable consisting of 11 binary indicators for 5-year age bracket intervals from age 16 to 70 years and one binary indicator for older than 70 years;
- sex: a binary variable indicating sex (0 for male; 1 for female);
- marital status: a factor variable consisting of four binary indicators for married, single, divorced, and widowed;
- number of family units in the household: number of household members;
- minority: a binary variable indicating whether an individual is an ethnic minority;
- natural log of individual income;
- natural log of household income;
- socioeconomic groups: 18 socioeconomic groups were identified according to the GHS (36); and
- education: a factor variable consisting of 4 indicators for the highest educational qualification achieved (3 for degree, 2 for A-levels or equivalent, 1 for O-levels or equivalent, 0 for no qualification).

Equation 1 indicates that smoking may be a function of illness; however, it is also possible that the opposite is true. That is, Equation 2 shows how illness may be a function of smoking. That is, not only does this group of individuals tend to smoke, but smoking may also cause long-term illness, disability, or infirmity, as discussed previously (37–40).


(2) $$\begin{array}{l} Illness_{i}=c_{2}+\alpha_{2}Smoking_{i}+\beta_{2}X_{i}^{{}^{\prime }}+\epsilon_{2,i} \end{array}$$

We decided to implement an instrumental variable regression to rule out this simultaneous effect (41). We used the number of vehicles owned as the instrumental variable. This instrument was chosen because individuals with long-term illness, disability, and infirmity are less able to drive, resulting in them being less likely to purchase vehicles and their family owning less vehicles (42–44). The relevance condition is testable; the rule of thumb is that if *F* > 10, then the relevance condition is satisfied, meaning that there is a statistically significant association between the instrument (**Vehicle ownership**) and explanatory variable (**Illness**). The exogeneity assumption is not testable, but it should be satisfied given our all-around controls denoted by X' to avoid potential omitted variables that may result in biased OLS estimators. That is, the instrument is uncorrelated with the error term, and only influences smoking status indirectly through illness. Therefore, our first-stage equation is the following:


(3) $$\begin{array}{l} Illness_{i}=c_{3}+\alpha_{3}Vehicle_{i}^{\prime }+\beta_{3}X_{i}^{{}^{\prime }}+\epsilon_{3,i} \end{array}$$

where **Vehicle**' denotes a vector consisting of the dummies of the vehicles owned. We used the predictions of illness $\hat{Illnesss_{i}}$ as part of the second-stage equation in an instrumental variable specification:


(4) $$\begin{array}{l} Smoking_{i}=c_{4}+\alpha_{4}\hat{Illnesss_{i}}+\beta_{4}X_{i}^{{}^{\prime }}+\epsilon_{4,i}. \end{array}$$

We firstly chose to use a linear probability model (LPM) in both stages as our “baseline” regression (24). Because both smoking and illness are binary variables, we further used recursive bivariate probit model (RBPM) where we used probit model in both stages to enhance the consistency of results. Generally, we prefer RBPM model because it is designed for binary outcomes (e.g., both smoking and LSI have only two possible outcomes). We additionally implemented the endogeneity test (Table 3), which reported the endogeneity of the illness variable, again indicating the need to implement instrumental variable regression. The null hypothesis is that the specified endogenous regressors can actually be treated as exogenous. This test is a robustness check against our claim of the need for an IV regression: an insignificant test statistic indicates that generic OLS regression (Equation 1) is sufficient, and IV regression is unnecessary. All analyzes were completed in Stata 16.

**Table 3:** Instrumental variable (IV) specifications.

	**LPM**		**RBPM**	
	**First-stage** **(Equation 3)**	**Second-stage** **(Equation 4)**	**First-stage** **(Equation 3)**	**Second-stage** **(Equation 4)**
Illness	–	0.90^***^ (5.74)	–	0.28^***^ (6.96)
**# of vehicles owned**				
At least 1	−0.06^***^ (−4.22)	–	−0.06^***^ (−4.16)	–
At least 2	−0.11^***^ (−6.91)	–	−0.11^***^ (−6.69)	–
At least 3	−0.14^***^ (−7.13)	–	−0.14^***^ (−6.81)	–
Additional controls	Yes	Yes	Yes	Yes
Demographic controls	Yes	Yes	Yes	Yes
Socioeconomic controls	Yes	Yes	Yes	Yes
**Regression statistics**				
*N*	12,297	12,297	12,297	12,297
*F*	21.71	–	60.89	–
Probability > *F*	0.00	0.00	0.00	0.00
IV endogeneity test	–	Yes^***^ (64.918)	–	Yes^***^ (32.12)

^*^p < 0.10.

^**^p < 0.05.

*** *p* < 0.01.

*t*- and chi-statistics are presented in parentheses.

## 3. Results

Table 3 presents the results of our instrumental variable specifications with both first-stage and second-stage equations. For IV specification LPM, in the first-stage equation, significant and negative results are reported for at least 1 vehicle owned (−0.06, *t* = −4.22), at least 2 vehicles owned (−0.11, *t* = −6.91), and at least 3 vehicles owned (−0.14, *t* = −7.13), with 0 vehicles owned as the reference group. In the second-stage equation, the main outcome of interest in the instrumental variable specification, a positively large causal impact of long-term illness, disability, or infirmity on the possibility of smoking, is reported (0.90, *t* = 5.74). The *F* statistic in Table 3 indicated the validity of our instruments. For IV specification RBPM, in the first-stage equation, significant and negative results are reported for at least 1 vehicle owned (−0.06, *t* = −4.16), at least 2 vehicles owned (−0.11, *t* = −6.69) and at least 3 vehicles owned (−0.14, *t* = −6.81), with 0 vehicles owned as the reference group. In the second-stage equation, the main outcome of interest in the instrumental variable specification, a positively large causal impact of long-term illness, disability, or infirmity on the possibility of smoking, is reported (0.28, *t* = 6.96). The *F*-statistic is 60.89 in the first-stage equation, demonstrating the validity of our claimed relevance condition.

## 4. Discussion

This study intended to find the causal impact of being long-term ill, disabled, or infirm on the status of smoking. Our study implemented an instrumental variable regression on a linear probability model and recursive bivariate probit model. We used a heteroskedastic version of the regression model for robustness. In the first-stage regression, we found negative associations between having 1, 2 or 3+ vehicles relative to having none and long-term illness. This supports our claim that individuals with long-term disease, disability, or infirmity are less likely to drive or not allowed to drive and therefore own fewer vehicles. Alternatively, the association may also indicate that individuals with more vehicles are less likely to have long-term illness, disability, or infirmity. The second-stage regression reported that individuals with long-term illness, disability, or infirmity were 28% more likely to smoke. As in IV regressions the estimates only include unidirectional effect, this study empirically proves that individuals with long-term illness, disability, or infirmity are more probably to smoke comparing to healthy individuals.

Admittedly, our findings have some limitations. Although we estimated a positive causal impact of LSI on smoking, there is insufficient quantitative data and evidence for us to disentangle the reasons why individuals with long-term illnesses are more likely smokers. One possible explanation is that smoking could be a method of coping with psychological issues due to long-term illness; studies have found that tobacco consumption can control stress levels, alleviate signs of depression, and enhance wellbeing in the short term (45, 46). Although smoking may be a way to ease anxiety and distress in the short term for people with chronic mental health issues, no concrete research suggests that smoking has a positive influence on subjective mental health over the long term (12, 47–49). Additionally, disabled persons generally experience greater discrimination than the general population. These perceived discriminations are also connected with increased stress and a higher likelihood of tobacco use (50, 51). However, the extent to which these factors affect the smoking rate remains quantitatively unclear, warranting further investigation in the future.

Also, the cohort of our studies is only UK residents, implying that it may only identify a local treatment effect of having disability, infirmity, and long-term illness. That is, this causal effect found by our studies may be different in another country, such as those “south countries” (41). Further, another potential limitation is caused by the self-reported data since individuals might provide a socially acceptable response rather than the truth. Given that smoking may not be regarded as an appropriate practice in certain places, respondents may conceal their smoking status by responding that they do not smoke. However, as the data collection is anonymous and we have used the instrumental variable framework, the selection bias is largely eliminated. In addition, a potential relationship between car ownership and smoking might exist irrespective of LSI status, as car ownership could be an indicator of socio-economic status, violating the IV exclusion assumption. Although this relationship is not testable, this is less of a problem as we have a rich set of controls, including income and socio-economic status.

Despite these limitations, this study can inform social care workers, psychologists, and public health practitioners to take more care of the health state of this marginalized group (individuals with LSI). Due to their current health state, they may face subtle or overt discrimination and have smaller networks, making them anxious and lonely. Regardless of why these individuals smoke more, the bidirectional relationship between smoking and illness creates a vicious cycle. So, to discourage smoking and improve health levels, the most common measure implemented by healthcare workers previously was not sufficient as those with the chronic illness are much more likely to be smokers. As such, it is necessary to take further care of those individuals and also target them directly by interventions to break the vicious cycle, which may alleviate the burden on National Health Service (NHS) hospitals (13).

This study fills a gap in the current literature, which includes only theoretical studies by psychologists (39) and lacks empirical studies due to the simultaneous effect. Our studies confirm this association as a causal relationship; that is, individuals with chronic disease are 28% more probable to be smokers, ceteris paribus. The present data suggest that physical impairments increase smoking, and if psychological problems play a role, it may be helpful for doctors and psychologists to target those processes. As such, the smoking frequency of disabled persons will decline, leading them to have better health, a greater life expectancy, and a lower burden of health expenditures (52).

In the future, we aim to extend our research to other countries, where people with disabilities, infirmities, and long-term illnesses have fewer social-care resources, and to compare how the results vary. We also aim to investigate the psychological reason that individuals with chronic diseases smoke more. Currently only psychological studies are discussed theoretically, and we aim to explain the theories empirically in future studies.

## 5. Conclusion

This study estimated the causal impact of long-term illness, disability, or infirmity on individual smoking behavior using the instrumental variable strategy on UK General Household Survey data. Although the descriptive statistics did not reveal a statistically significant correlation between long-term illness, disability, and individual smoking behavior, our instrumental variable estimates showed that individuals' long-term impairments could lead to a sizable smoking tendency.

The public policy implications of these findings are, to some extent, straightforward. Because people with disabilities and chronic diseases are more likely to be smokers, conventional targeted smoking cessation interventions were not very effective. In other words, we have been trying to address the result rather than the cause of smoking, so it might be best redirecting intervention effects toward individuals with long-term impairment. Other than conventional smoking cessation programs, policies for disabled people should also consider filling the inequalities between them and healthy individuals, such as providing accessible information for persons with cognitive impairments, promoting equal opportunities for jobs, and having accessible facilities or technologies readily available (53, 54).

## Data availability statement

The datasets presented in this study can be found in online repositories. The names of the repository/repositories and accession number(s) can be found in the article/supplementary material.

## Author contributions

XZ and YL conceived, designed the analysis, and collected the data. XZ, YL, and YX contributed to the data or analysis tools and performed the analysis. XZ, YL, TZ, and YX wrote the paper. All authors contributed to the article and approved the submitted version.

## References

[1] Centers for Disease C Prevention. Tobacco use—United States, 1900–1999. JAMA. (1999) 282:2202. 10.1001/jama.282.23.2202 10605963

[2] Holahan CK Holahan CJ North RJ Hayes RB Powers DA Ockene JK. Smoking status, physical health-related quality of life, and mortality in middle-aged and older women. Nicotine Tob Res. (2013) 15:662–9. 10.1093/ntr/nts182 22965789 PMC3611990

[3] King BA Dube SR Tynan MA. Current tobacco use among adults in the united states: findings from the National Adult Tobacco Survey. Am J Public Health. (2012) 102:e93–100. 10.2105/AJPH.2012.301002 22994278 PMC3477973

[4] Office of the Surgeon General USPHS. Smoking Cessation: A Report of the Surgeon General. (2020). p. 9–15. Available online at: https://www.hhs.gov/sites/default/files/2020-cessation-sgr-full-report.pdf

[5] Brawarsky P Brooks DR Wilber N Gertz RE Klein Walker D. Tobacco use among adults with disabilities in Massachusetts. Tob Control. (2002) 11 Suppl 2:ii29–33. 10.1136/tc.11.suppl_2.ii29 12034978 PMC1766081

[6] Armour BS Campbell VA Crews JE Malarcher A Maurice E Richard RA. State-level prevalence of cigarette smoking and treatment advice, by disability status, United States, 2004. Prev Chronic Dis. (2007) 4:A86. 17875261 PMC2099284

[7] Hall AG Schumacher JR Cannell MB Berry JB Schiaffino M Park S. Tobacco use in Florida: comparisons between adults living with and without disabilities. Disabil Health J. (2013) 6:213–9. 10.1016/j.dhjo.2013.01.008 23769480

[8] Niaura RS Rohsenow DJ Binkoff JA Monti PM Pedraza M Abrams DB. Relevance of cue reactivity to understanding alcohol and smoking relapse. J Abnorm Psychol. (1988) 97:133–52. 10.1037/0021-843X.97.2.133 3290304

[9] Tang R Razi A Friston KJ Tang Y-Y. Mapping smoking addiction using effective connectivity analysis. Front Hum Neurosci. (2016) 10:195. 10.3389/fnhum.2016.00195 27199716 PMC4854896

[10] Becker GS Murphy KM. Are addicts rational? A theory of rational addiction. Math Soc Sci. (1988) 16:217–8. 10.1016/0165-4896(88)90060-1

[11] Casseus M Graber JM West B Wackowski O. Tobacco use disparities and disability among US college students. J Am Coll Health. (2020) 7:1–6. 10.1080/07448481.2020.1842425 33151844

[12] Kaliterna Lipovčan L Brkljačić T Tadić M. Tobacco consumption, alcohol intake frequency and quality of life: results from a nationally representative Croatian Sample Study. Drus Istraz. (2013) 22:627–49. 10.5559/di.22.4.04

[13] Centers for Disease C, Prevention. Current Cigarette Smoking Among Adults in the United States. Washington, DC (2022).

[14] Fitzmaurice C Kanarek N Fitzgerald S. Primary prevention among working age USA adults with and without disabilities. Disabil Rehabil. (2011) 33:343–51. 10.3109/09638288.2010.490869 20524841

[15] Jarrett T Pignataro RM. Cigarette smoking among college students with disabilities: National College Health Assessment II, fall 2008–spring 2009. Disabil Health J. (2013) 6:204–12. 10.1016/j.dhjo.2013.01.011 23769479

[16] Pharr JR Bungum T. Health disparities experienced by people with disabilities in the United States: a behavioral risk factor surveillance system study. Glob J Health Sci. (2012) 4:99–108. 10.5539/gjhs.v4n6p99 23121746 PMC4776960

[17] Emerson E. Smoking among adults with and without disabilities in the UK. J Public Health. (2018) 40:e502–9. 10.1093/pubmed/fdy062 29617853

[18] Lee J-E Park J-H Kim H-R Shin H-I. Smoking behaviors among people with disabilities in Korea. Disabil Health J. (2014) 7:236–41. 10.1016/j.dhjo.2013.11.002 24680053

[19] Heydarpour P Manouchehrinia A Beiki O Mousavi SE Abdolalizadeh A Lakeh MM. Smoking and worsening disability in multiple sclerosis: a meta-analysis. Acta Neurol Scand. (2018) 138:62–9. 10.1111/ane.12916 29542102

[20] Wüst RCI Morse CI de Haan A Rittweger J Jones DA Degens H. Skeletal muscle properties and fatigue resistance in relation to smoking history. Eur J Appl Physiol. (2008) 104:103–10. 10.1007/s00421-008-0792-9 18560879 PMC2480601

[21] Zhang QC Courtney-Long EA Sinclair LB Reese S Armour BS Shapira SK. State-specific prevalence of current e-cigarette use by disability status and disability type—United States, BRFSS 2016–2018. Disabil Health J. (2022) 15:101182. 10.1016/j.dhjo.2021.101182 34391714 PMC8678284

[22] Feng Q Wu GL. On the reverse causality between output and infrastructure: the case of China. Econ Model. (2018) 74:97–104. 10.1016/j.econmod.2018.05.006

[23] Spearing NM Connelly LB Nghiem HS Pobereskin L. Research on injury compensation and health outcomes: ignoring the problem of reverse causality led to a biased conclusion. J Clin Epidemiol. (2012) 65:1219–26. 10.1016/j.jclinepi.2012.05.012 23017639

[24] Angrist JD Pischke JS. Mostly Harmless Econometrics: An Empiricist's Companion. Princeton, NJ: Princeton University Press (2009). p. 392.

[25] King JLMSCHES Pomeranz JLPDCRCCLCP Young MEPD Moorhouse MPDCRC Merten JWPDMCHES. Evaluation of a newly developed tobacco cessation program for people with disabilities. Disabil Health J. (2016) 9:145–9. 10.1016/j.dhjo.2015.08.002 26365086 PMC4688053

[26] Borrelli B Busch AM Trotter DRM. Methods used to quit smoking by people with physical disabilities. Rehabil Psychol. (2013) 58:117–23. 10.1037/a0031577 23437992 PMC3667967

[27] Schroeder SA Morris CD. Confronting a neglected epidemic: tobacco cessation for persons with mental illnesses and substance abuse problems. Annu Rev Public Health. (2010) 31:297–314. 10.1146/annurev.publhealth.012809.103701 20001818

[28] Baiocchi M Cheng J Small DS. Instrumental variable methods for causal inference. Stat Med. (2014) 33:2297–340. 10.1002/sim.6128 24599889 PMC4201653

[29] Leszczensky L Wolbring T. How to deal with reverse causality using panel data? Recommendations for researchers based on a simulation study. Sociol Methods Res. (2022) 51:837–65. 10.1177/0049124119882473

[30] Gelman A Imbens G. Why ask Why? Forward Causal Inference and Reverse Causal Questions. National Bureau of Economic Research (2013). 10.3386/w19614

[31] Becker SO. Using Instrumental Variables to Establish Causality. IZA World Labor (2016). 10.15185/izawol.250

[32] Darcy S Burke PF. On the road again: the barriers and benefits of automobility for people with disability. Transp Res Part A Policy Pract. (2018) 107:229–45. 10.1016/j.tra.2017.11.002

[33] Ali R Greer J Matthews D Murray L Robinson S Sattar G. General Household Survey 2006 Definitions and Terms. Newport, RI (2008).

[34] Nasir R Brookman A. Smoking Prevalence in the UK the Impact of Data Collection Changes: 2020. Office for National Statistics (2020). p. 1–5. Available online at: https://www.hhs.gov/sites/default/files/2020-cessation-sgr-full-report.pdf

[35] Bao Y Duan N Fox SA. Is some provider advice on smoking cessation better than no advice? An instrumental variable analysis of the 2001 National Health Interview Survey. Health Serv Res. (2006) 41:2114–35. 10.1111/j.1475-6773.2006.00592.x 17116112 PMC1955314

[36] Office for National Statistics. General Household Survey (2006). UK Data Service (2009). 10.5255/UKDA-SN-5804-1

[37] Lincoln AE Smith GS Amoroso PJ Bell NS. The effect of cigarette smoking on musculoskeletal-related disability. Am J Ind Med. (2003) 43:337–49. 10.1002/ajim.10195 12645091 PMC2141692

[38] Manouchehrinia A Tench CR Maxted J Bibani RH Britton J Constantinescu CS. Tobacco smoking and disability progression in multiple sclerosis: United Kingdom cohort study. Brain. (2013) 136:2298–304. 10.1093/brain/awt139 23757766 PMC3692034

[39] Macdonald SJ Deacon L Nixon J Akintola A Gillingham A Kent J. “The invisible enemy”: disability, loneliness and isolation. Disabil Soc. (2018) 33:1138–59. 10.1080/09687599.2018.1476224

[40] Bengtsson T Nilsson A. Smoking and early retirement due to chronic disability. Econ Hum Biol. (2018) 29:31–41. 10.1016/j.ehb.2017.12.005 29413586

[41] Angrist JD Imbens GW Rubin DB. Identification of causal effects using instrumental variables. J Am Stat Assoc. (1996) 91:444–55. 10.1080/01621459.1996.1047690223180483

[42] Clark B Lyons G Chatterjee K. Understanding the process that gives rise to household car ownership level changes. J Transp Geogr. (2016) 55:110–20. 10.1016/j.jtrangeo.2016.07.009

[43] Brumbaugh S. Travel Patterns of American Adults with Disabilities. Department of Transportation (2018). Available online at: https://rosap.ntl.bts.gov/view/dot/37593

[44] Etminani-Ghasrodashti R Ketankumar Patel R Kermanshachi S Rosenberger JM Foss A. Modeling users' adoption of shared autonomous vehicles employing actual ridership experiences. Transp Res Rec. (2022) 11:462–78. 10.1177/03611981221093632

[45] Weinhold D Chaloupka FJ. Smoking status and subjective wellbeing. Tob Control. (2017) 26:195–201. 10.1136/tobaccocontrol-2015-052601 27098008

[46] Prochaska JJ Das S Young-Wolff KC. Smoking, mental illness, and public health. Annu Rev Public Health. (2017) 38:165–85. 10.1146/annurev-publhealth-031816-044618 27992725 PMC5788573

[47] Smith CA McNeill A Kock L Shahab L. Exploring mental health professionals' practice in relation to smoke-free policy within a mental health trust: a qualitative study using the COM-B model of behavior. BMC Psychiatry. (2019) 19:54. 10.1186/s12888-019-2029-3 30717722 PMC6360690

[48] Saluja G Iachan R Scheidt PC Overpeck MD Sun W Giedd JN. Prevalence of and risk factors for depressive symptoms among young adolescents. Arch Pediatr Adolesc Med. (2004) 158:760. 10.1001/archpedi.158.8.760 15289248

[49] Parker MA Cordoba-Grueso WS Streck JM Goodwin RD Weinberger AH. Intersectionality of serious psychological distress, cigarette smoking, and substance use disorders in the United States: 2008–2018. Drug Alcohol Depend. (2021) 228:109095. 10.1016/j.drugalcdep.2021.109095 34601273 PMC8595675

[50] Schrempft S Jackowska M Hamer M Steptoe A. Associations between social isolation, loneliness, and objective physical activity in older men and women. BMC Public Health. (2019) 19:74. 10.1186/s12889-019-6424-y 30651092 PMC6335852

[51] Purnell JQ Peppone LJ Alcaraz K McQueen A Guido JJ Carroll JK. Perceived discrimination, psychological distress, and current smoking status: results from the behavioral risk factor surveillance system reactions to race module, 2004–2008. Am J Public Health. (2012) 102:844–51. 10.2105/AJPH.2012.300694 22420821 PMC3483933

[52] Yokota RTC Nusselder WJ Robine J-M Tafforeau J Charafeddine R Gisle L. Contribution of chronic conditions to smoking differences in life expectancy with and without disability in Belgium. Eur J Public Health. (2018) 28:859–63. 10.1093/eurpub/cky101 29901735

[53] Everding J Marcus J. The effect of unemployment on the smoking behavior of couples. Health Econ. (2020) 29:154–70. 10.1002/hec.3961 31820539

[54] Dicianno BE Sivakanthan S Sundaram SA Satpute S Kulich H Powers E. Systematic review: automated vehicles and services for people with disabilities. Neurosci Lett. (2021) 761:136103. 10.1016/j.neulet.2021.136103 34237416

